# Preclinical In Vivo-Models to Investigate HIPEC; Current Methodologies and Challenges

**DOI:** 10.3390/cancers13143430

**Published:** 2021-07-08

**Authors:** Roxan F. C. P. A. Helderman, Daan R. Löke, Pieter J. Tanis, Jurriaan B. Tuynman, Wim Ceelen, Ignace H. de Hingh, Kurt van der Speeten, Nicolaas A. P. Franken, Arlene L. Oei, H. Petra Kok, Johannes Crezee

**Affiliations:** 1 Center for Experimental and Molecular Medicine (CEMM), Laboratory for Experimental Oncology and Radiobiology (LEXOR), Amsterdam UMC, University of Amsterdam, Cancer Center Amsterdam, 1105 AZ Amsterdam, The Netherlands; f.c.helderman@amsterdamumc.nl (R.F.C.P.A.H.); n.a.franken@amsterdamumc.nl (N.A.P.F.); a.l.oei@amsterdamumc.nl (A.L.O.); 2 Department of Radiation Oncology, Amsterdam UMC, University of Amsterdam, Cancer Center Amsterdam, 1105 AZ Amsterdam, The Netherlands; d.r.loke@amsterdamumc.nl (D.R.L.); h.p.kok@amsterdamumc.nl (H.P.K.); 3 Department of Surgery, Amsterdam UMC, University of Amsterdam, Cancer Center Amsterdam, 1105 AZ Amsterdam, The Netherlands; p.j.tanis@amsterdamumc.nl; 4 Department of Surgery, Amsterdam UMC, Vrije Universiteit Amsterdam, 1081 HV Amsterdam, The Netherlands; j.tuynman@amsterdamumc.nl; 5 Department of GI Surgery, Ghent University Hospital, 9000 Ghent, Belgium; wim.ceelen@ugent.be; 6 Department of Surgery, Catharina Cancer Institute, 5602 ZA Eindhoven, The Netherlands; ignace.d.hingh@catharinaziekenhuis.nl; 7 GROW-School for Oncology and Developmental Biology, Maastricht University, 6200 MD Maastricht, The Netherlands; 8 Department of Surgical Oncology, Ziekenhuis Oost-Limburg, 3600 Genk, Belgium; kurt.vanderspeeten@zol.be

**Keywords:** peritoneal carcinomatosis, peritoneal metastasis, cytoreductive surgery (CRS), hyperthermic intraperitoneal chemotherapy (HIPEC)

## Abstract

**Simple Summary:**

Efficacy of cytoreductive surgery (CRS) combined with hyperthermic intraperitoneal chemotherapy (HIPEC) depends on patient selection, tumor type, delivery technique, and treatment parameters such as temperature, carrier solution, type of drug, dosage, volume, and treatment duration. Preclinical research offers a powerful tool to investigate the impact of these parameters and to assists in designing potentially more effective treatment protocols and clinical trials. This study aims to review the objectives, methods, and clinical relevance of in vivo preclinical HIPEC studies found in the literature. In total, 60 articles were included in this study. The selected articles were screened on the HIPEC parameters. Recommendations are provided and possible pitfalls are discussed on the choice of type of animal and tumor model per stratified parameters and study goal. The guidelines presented in this paper can improve the clinical relevance and impact of future in vivo HIPEC experiments.

**Abstract:**

Hyperthermic intraperitoneal chemotherapy (HIPEC) is a treatment modality for patients with peritoneal metastasis (PM) of various origins which aims for cure in combination with cytoreductive surgery (CRS). Efficacy of CRS-HIPEC depends on patient selection, tumor type, delivery technique, and treatment parameters such as temperature, carrier solution, type of drug, dosage, volume, and treatment duration. Preclinical research offers a powerful tool to investigate the impact of these parameters and to assist in designing potentially more effective treatment protocols and clinical trials. The different methodologies for peritoneal disease and HIPEC are variable. This study aims to review the objectives, methods, and clinical relevance of in vivo preclinical HIPEC studies found in the literature. In this review, recommendations are provided and possible pitfalls are discussed on the choice of type of animal and tumor model per stratified parameters and study goal. The guidelines presented in this paper can improve the clinical relevance and impact of future in vivo HIPEC experiments.

## 1. Introduction

Metastatic lesions in the peritoneum are commonly originating from primary malignancies such as ovarian, colorectal, appendiceal, and gastric cancer [1]. During hyperthermic intraperitoneal chemotherapy (HIPEC), a heated chemotherapeutic solution is circulated through the abdominal cavity immediately after cytoreductive surgery (CRS). CRS aims to eradicate all macroscopically visible tumor lesions present in the peritoneal cavity, HIPEC is given to eradicate any remaining microscopic disease. The combination with CRS and HIPEC has shown prolonged overall survival compared to CRS alone and systemic chemotherapy-based treatments [2,3,4]. The recently randomized trial performed by van Driel et al. showed a median overall survival of 45.7 months for ovarian cancer patients with peritoneal metastasis (PM) treated with CRS and cisplatin-based HIPEC, which was significantly higher than the median 33.9 months for CRS alone [5]. The relevance of a careful choice of treatment parameters was recently underscored by the outcome of the PRODIGE-7 trial in which patients were treated with high-dose oxaliplatin by the closed (360 mg/m^2^) or open (460 mg/m^2^) delivery technique at a relatively high temperature (43 °C) for a short (30 min) duration [6]. The trial showed no survival benefit and increased morbidity in the CRS plus HIPEC arm, compared to CRS alone. This opened up the debate on the benefit of HIPEC, specifically for patients with PM from colorectal origin. Some surgeons argued that this trial proved that the optimal treatment strategy is CRS without HIPEC [7]. Others argued that the treatment parameters used during the PRODIGE-7 trial were not adequately chosen and therefore, that the results cannot be regarded as representative of HIPEC in general [8,9,10]. The majority of the included patients were treated with induction systemic chemotherapy and only responders were selected for CRS with or without HIPEC. In a comment, Ceelen argued that the choice of chemotherapy, treatment duration, carrier solution, and treatment temperature could have negatively impacted trial outcomes [8]. Not all colorectal cancer subtypes respond similarly to oxaliplatin and the treatment duration was not optimal to maximize the effect of oxaliplatin exposure [11]. Dextrose 5% was chosen as the carrier solution, possibly resulting in hyponatremia and hyperglycemia. High dextrose can also change the macro-environment across the peritoneal surface. Lastly, the performed surgery might increase the sensitivity of the peritoneal surface to high temperatures and associated thermal toxicity, possibly increasing morbidity. Besides pharmacological weak spots, the PRODIGE-7 trial is argued to have four more design shortcomings and should thus not be used to discredit all other HIPEC regimes [12]. In a recent review by Auer et al., evidence-based indications were investigated for the application of HIPEC and CRS for patients diagnosed with mesothelioma, appendiceal, colorectal, gastric ovarian, and primary peritoneal carcinoma. It was concluded that there was enough evidence for recommending HIPEC for the treatment of newly diagnosed, primary stage III epithelial ovarian, fallopian or primary peritoneal carcinoma when CRS was complete. Present clinical evidence was deemed insufficient for HIPEC for PM of other origins, these patients should be treated within study protocols to collect further evidence [13]. This clinical evidence is vital for successful application of HIPEC and therefore, these study protocols should be designed to be able to provide solid recommendations on optimal treatment.

Strong scientific evidence for the selection of treatment parameters is essential to optimize treatment protocols and design successful clinical trials. The efficacy of the HIPEC procedure may be influenced by patient selection, delivery technique, and treatment parameters, including temperature, carrier solution, type of drug, dosage, volume, and treatment duration [14]. Lack of sufficient data may explain why the choice of these parameters varies significantly among HIPEC experts around the world [15]. Therefore, solid scientific evidence is needed to support an optimal choice of treatment parameters.

Several factors associated with treatment effectiveness have already been evaluated in clinical studies. The small-bowel-peritoneal cancer index (PCI) score was shown to be a valuable prognostic factor for overall survival in the retrospective analysis [16]. Spielberg et al. showed in another retrospective study in patients suffering from PM of colorectal cancer origin that the use of oxaliplatin resulted more frequently in postoperative complications compared to MMC [17]. A study in gastric cancer patients showed that complete CRS is important for survival [18], while lymph node involvement has a negative effect on progression-free survival [19]. Fagotti et al. showed that recurrent ovarian cancer patients could be safely treated with minimally invasive surgery in combination with cisplatin- or oxaliplatin-based HIPEC, showing promising results [20,21]. However, experimentation during clinical studies is generally limited and more often retrospective.

Preclinical research offers a powerful tool for investigating the impact of HIPEC treatment parameters on treatment outcomes. Data provided by in vitro research can provide interesting insights into the molecular effect of various chemotherapeutic agents on tumor and normal tissue cell lines at different, well-controlled, temperatures [22,23,24]. Recently, Ubink et al. created organoids derived from peritoneal metastases from patients to initiate a preclinical platform to evaluate HIPEC regimes [25]. Although experiments performed on monolayer, 3D cell cultures, and organoids do have specific advantages, they do not accurately reflect the complexity of a patient. Therefore, they are not considered the most representative models to study clinical HIPEC conditions. Two major limitations in translating results of in vitro cell cultures to the clinic are the absence of a tumor microenvironment and the lack of normal tissue with corresponding systemic phenomena including pharmacokinetics and pharmacodynamics. Adequate in vivo models thus remain essential for the successful translation of preclinical results into relevant clinical protocols. Such HIPEC models should meet a number of requirements. First of all, models should reflect the range of possible choices made before and during clinical HIPEC treatments as much as possible. Furthermore, it should be possible to vary individual treatment parameters while keeping other parameters constant to allow differentiation between the effects of different treatment parameters on the efficacy of HIPEC.

**Figure 1:**
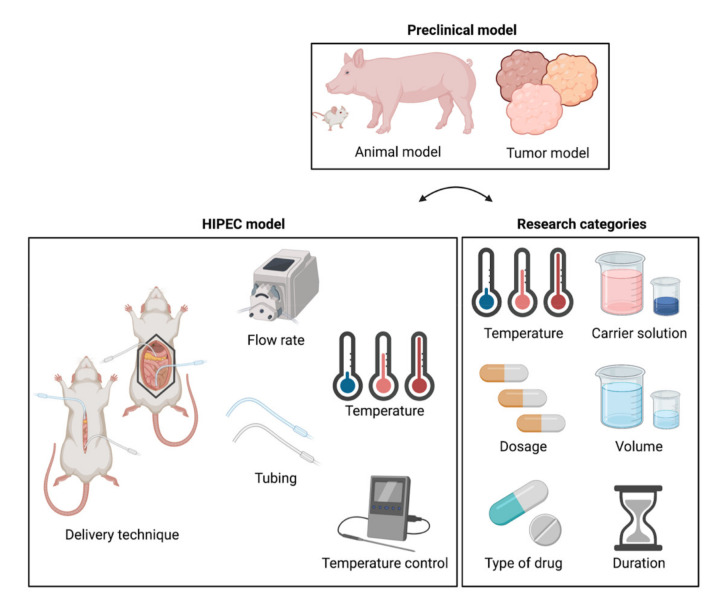
Overview of important parameters for performing HIPEC in preclinical models. The preclinical model consists of a suitable animal model with a corresponding tumor model. The delivery technique, flow rate, tubing setup, temperature, and temperature control are relevant for the HIPEC model. The research categories are HIPEC treatment parameters with an impact on the treatment outcome; i.e., temperature, carrier solution, dosage, volume, type of drug, and duration.

In recent years, a large number of in vivo HIPEC studies have been performed with various objectives and methods. These studies all have advantages and disadvantages, depending on the aim of the study. There are many general HIPEC review papers in which also preclinical models are discussed [26,27,28,29,30,31], but so far, no comprehensive review discussing these preclinical in vivo models for HIPEC is available, and such a specific review will assist in making optimal model choices for in vivo research. This study reviews the objectives, methods, advantages, and limitations of preclinical animal models used for research on relevant in vivo HIPEC treatment parameters relevant for treatment outcome. Relevant features and challenges of preclinical animal models, the HIPEC setup, and research categories are discussed (Figure 1). Finally, guidelines are provided on how to design and develop models specifically aiming at certain parameters.

## 2. Methods

This review is structured as a list of relevant HIPEC parameters, where the model requirements are discussed for each treatment parameter, illustrated by discussing preclinical in vivo models from the literature. The latter were identified by performing a literature search on PubMed in March 2021. Search terms that were used included “HIPEC”, “Pigs”, “Rabbits”, “Porcine”, “Mice”, “Murine” and “Rats”. Only in vivo research articles written in English were included. In total, 60 articles were included in this study, published between 1998 and 2020. The selected articles were screened on the following parameters: type of animal, tumor model, type of delivery, number of inflows/outflows, flow rate, treatment duration, volume of the perfusate, type of carrier solution, type of drug, dosage, temperature of the perfusate, availability thermal measurements, core temperature, type of cooling/heating, flushing after HIPEC, surface area calculation method and goal of the study. These parameters and all studies are listed and cited in the supplementary material as well.

## 3. HIPEC Research Categories

The 60 in vivo HIPEC research papers discussed in this review investigated a wide range of research questions. These research questions are organized into research categories (see Table 1). We discuss the studies performed per research category, possible future experiments essential for optimizing HIPEC, what is needed to ensure adequate performance, and possible pitfalls. Additionally, we discuss crucial requirements for representative and safe in vivo HIPEC research resulting in thermal homogeneity and prevention of overheating. A full overview of the studies included and the investigated treatment parameters are provided in Supplementary Table S1. In Table 1 the research categories, the number of studies in each research category, and recommendations for tumor and animal models for future studies are presented. In the remainder of this review, the choices listed in Table 1 are explained and discussed.

## 4. Preclinical HIPEC Models

Important parameters of preclinical HIPEC research models to study physiological effects and anticancer activity include a selection of a suitable animal model, tumor model, and setup. We can distinguish two types of models: physiological models, which determine for example tolerable temperatures and volumes to establish whether a drug can be given in the peritoneum, and anticancer models to evaluate the expected level of anticancer activity. The selection of relevant parameters depends on the research category and study goals. Considerations for proper selection are discussed in separate sections for each of these research model parameters, followed by sections on each of the research categories.

### 4.1. Animal Model

Several types of animals have been used as a preclinical model to study HIPEC in the analyzed articles. Most often rats (47%) were used, followed by mice (27%), pigs (22%) and rabbits (5%). For all animals, both male and female animals were used during the HIPEC procedures. Depending on the origin of the used cancer cells, rat and mouse strains were chosen. The following rat types were used in the analyzed articles; Wistar (21%) [32,45,46,62,63,64], Sprague Dawley (32%) [40,43,51,52,57,65,66,67,68], WAG/Rij (32%) [47,54,69,70,71,72,73,74,75] and athymic/nude (14%) [44,76,77,78] rats. For mouse experiments, the following strains were used: Swiss albino (20%) [36,38,79], athymic (33%) [37,48,50,55,80], C57BL/6 (20%) [33,39,81], BALB/c (20%) [49,56,82] and NOD-SCID (7%) [34]. The following pig strains were used: white (38%) [58,60,61,83,84], sus scrofa domesticus (23%) [85,86,87], mini (8%) [59] and pigs without specifying the strain (31%) [88,89,90]. New Zealand white rabbit was the only used rabbit strain [41,42,91].

The main advantage of using small animals in preclinical HIPEC studies is the fact that one can relatively easily establish PM from a variety of origins. The main drawback of using small animals is the significantly smaller peritoneal cavity compared to a human’s. To that end, the physiological parameters, such as volume, tolerated temperature of the heated solution, carrier solution, pharmacodynamics, and pharmacokinetics are less representative of the human situation. In general, small animal models can only be used to study the local effects of HIPEC and are most suitable to study anticancer effects such as the tumor penetration depth, tumor biology, tumor sensitivity, and tumor survival. This includes the determination of the tumor-specific optimal treatment temperature, with the additional macro-environment providing interesting insights compared to 2D cell cultures used in in vitro studies. Because of the smaller size of the peritoneal cavity and the small body size, affecting the pharmacodynamics of the chemotherapy, systemic phenomena are difficult to directly translate to human clinical settings. This includes pharmacokinetics, pharmacodynamics, and side effects. The small flow region is also not ideally suited to test the optimal clinical flow setup where thermal and drug distributions can vary widely between flow region sizes. Large animals are more suitable to use when investigating these specific research questions.

**Figure 2:**
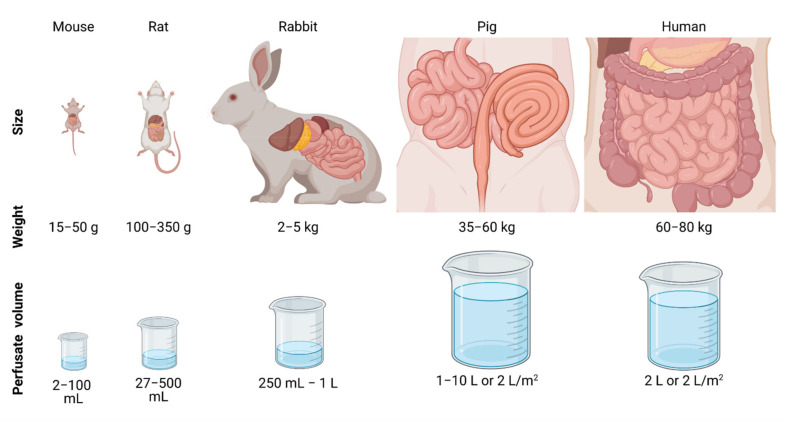
Schematic overview of the type of animals to present the size, weight, and perfusate volume applied during the HIPEC procedures.

Large animals, such as pigs, were less frequently used as a preclinical model in HIPEC research. This could be due to higher costs, the requirement of specific housing, and the lack of trained personnel. More importantly, there are currently no relevant PM pig models available. The only reported PM pig model was established using a human cervix cancer human cell line [92]. Lack of good tumor models in pigs, i.e., development of tumor lesions in the peritoneal area, hamper evaluation of the effect of HIPEC effects on tumor size or survival. Nevertheless, larger animal models are ideal for studying the macroscopic and systemic effects of HIPEC, because both size and anatomy are comparable to the human peritoneum, see Figure 2. This is especially important when studying temperature distribution, drug distribution, pharmacokinetics, pharmacodynamics, and side effects. Determining these factors in smaller animals is also possible, but the clinical translation would be more complex, and therefore, larger, preferably more human-size animals are preferred. The feasibility and safety of innovations regarding the flow setup can be tested in large animals to allow reliable translation to the clinic.

### 4.2. Tumor Model

PM were induced in most of the reviewed articles (52%), while 48% of the studies performed HIPEC in animals when no PM were induced. In the included articles, PM was induced in rats (46%), mice (100%) and rabbits (66%) before HIPEC treatment. Most PM models were syngeneic (69%) [32,33,35,36,38,39,41,42,44,45,47,49,54,56,64,69,70,72,73,74,79], whereas xenograft (28%) [34,37,48,50,55,78,80,81] and patient derived xenograft (PDX) (3%) [77] models were used in only 9 of the 29 used models. Tumors were established from different cell lines originating from colorectal (45%) [39,44,47,49,50,54,55,56,69,70,72,73,74,82], ovarian (29%) [32,33,37,45,48,64,78,80,81], breast (10%) [35,36,79], gastric (3%) [34], skin (3%) [38] cancer and pseudomyxoma peritonei (3%) [77].

**Figure 3:**
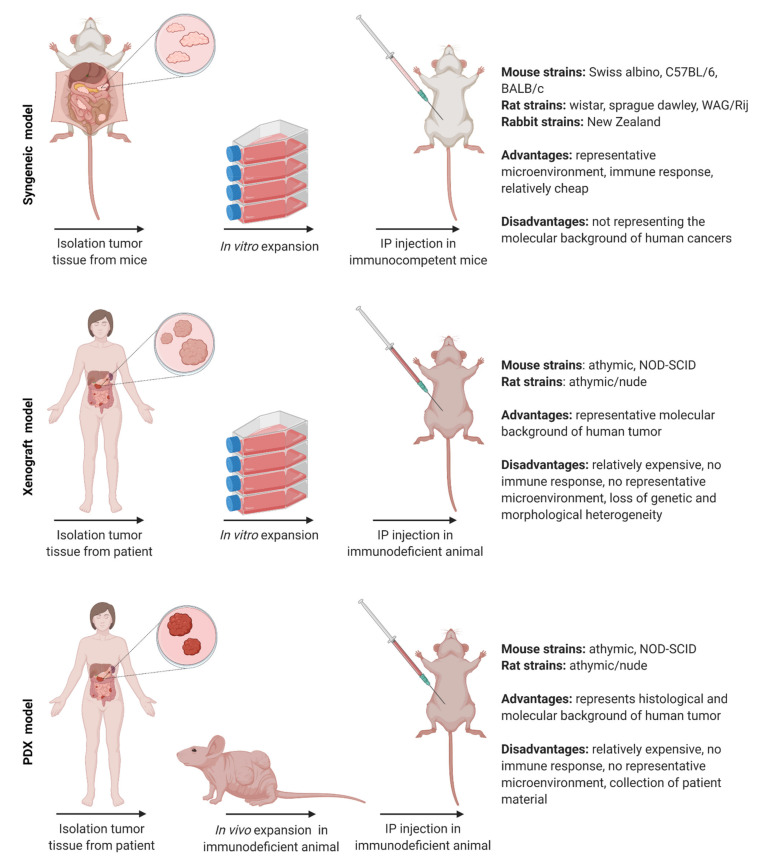
Schematic representation of the different tumor models with the according animal strains and their advantages and disadvantages.

Animals were injected with cancer cells, or cancer tissue was transplanted into the peritoneal area to develop small tumor lesions throughout the abdominal cavity. The models can be subdivided into three groups: syngeneic, xenograft, and PDX models (Figure 3). Syngeneic models are immunocompetent animals that receive cancer cells or tissue derived from the same genetic background [93]. The advantage of this model is the representative microenvironment. However, syngeneic models do not replicate the molecular background of human cancers. Xenograft models use immunodeficient animals with human cancer cells or tissue introduced in the peritoneum. Several immunodeficient mouse and rat strains are available, which lack mature T-lymphocytes, often in combination with the lack of B-lymphocytes and/or natural killer cells. PDX models are immunodeficient animals in which patient-derived cancer cells or tissues are grafted to overcome the loss of genetic and morphological heterogeneity of the xenograft model. This model represents the histological and molecular properties of the originating human clinical material obtained from patients. The use of PDX models is limited since it is complicated to obtain patient material and to establish patient-derived tumors in animals. Furthermore, immunodeficient animals are relatively expensive. However, even with these disadvantages, the PDX model remains the most clinically relevant and translatable preclinical model.

Each tumor model has its advantages which should be taken into account. All tumor models can be used to study the anticancer activity such as tumor penetration depth of the drug, but xenograft and PDX models should be used to study the tumor biology, excluding immune-related phenomena, and tumor sensitivity. When applying immunotherapy or studying the immune response, the syngeneic model is a logical choice.

Only in half of the studies (31 out of 60) HIPEC was performed in an animal model with PM, which underlines that the development of PM in animal models is a major challenge. Studies in which PM was successfully developed used different cell lines, cell amount, and dissolvent, resulting in the corresponding tumor take rates, tumor outgrowth time, PCI score, and tumor locations. This information is summarized in Table 2. Overall, the tumor implementation-take rate was 64–100%, with tumor formation seen from 2–27 days after injection, and a PCI score of 2–20, depending on the tumor model. In rats, the colorectal cancer cell line CC531 was most often used, with a tumor take rate of 100%, tumor outgrowth seen at 7–8 days after injection, resulting in tumor nodules mainly present on the greater omentum, liver hilum, perisplenic area and mesentery [47,54,69,70,72,73,74]. PM originating from ovarian cancer cells was also established in rats, mainly resulting in ascites, visceral and parietal peritoneum [32,45,64]. In mice, HCT116 [50,55], EAT [36,79] and CT26 [49,56] cells were used to establish PM. In rabbits, PM of gastric origin was established by injecting VX2 cells in the stomach of the animals [41,42]. The VX2 cell lines are derived from an oncogenic DNA virus of the Papovaviridae family and result in malignant skin lesions [94], resulting in many small (0.5–1.0 cm), hard and transparent tumor nodules developed on the greater omentum and the antrum of the stomach in 100% of the animals nine days after inoculation [41,42]. Unfortunately, the majority of the analyzed studies lack relevant information on the development of PM. High-resolution pictures showing the spread of the tumor nodules in the peritoneum and a proper scoring (e.g., PCI score) are often missing.

### 4.3. Experimental HIPEC Model

The concept of HIPEC is to penetrate any potential tumor nodules with a sufficient amount of chemotherapy anywhere at the peritoneal surface, which requires an equal distribution of the perfusate with the same temperature and concentration. In current HIPEC models, this desired equal distribution is not necessarily the case. Controlling and stabilizing the thermal profile is a difficult task. This was underlined by a study that measured thermal profiles in rats during a semi-open HIPEC, showing that fluctuations of a few degrees are not uncommon [95]. Three flow parameters can be adjusted to optimize the thermal homogeneity during open and close HIPEC treatments: flow rate, number of catheters, and catheter placement.

The flow rate can impact both the heating rate and homogeneity during treatment [95]. The ideal flow rate is highly dependent on the type of animal. The peritoneal volume, i.e., the total heat applied to the system, is related to the cubed length scale while the surface area, i.e., the cooling rate of the animal, depends on the squared length scale. The ratio of the heat applied and the cooling rate is therefore proportional to the length scale, making it relatively more difficult to reach the required treatment temperature within the peritoneal cavity. Ideal flow rates vary between animal species, and should ideally be determined per type of animal. In a previous study, HIPEC treatments in rats were simulated at various flow rates. Doubling the flow rate increased the thermal homogeneity while halving the flow rate resulted in increased thermal heterogeneity [76,95]. Higher flow rates tend to increase the homogeneity, but also the core temperature. Maximizing flow rates, while maintaining a tolerable core temperature should be preferred. Increasing the number of inflows catheters from 1 to 4 increased the thermal homogeneity and the stability of inflow temperatures in rats [76]. The placement of the catheters can also increase the distribution of the heated fluid. Maximizing the distance between the inflow and outflow catheters can also increase homogeneity. The maximum number of catheters that fit in the rat is 5. Similar studies are needed to determine the ideal number and placement of catheters for different animals.

The choice of inflow/outflow tubing in studies considered was fairly basic. Almost all evaluated studies featured one inflow and one outflow. Only one study considered more catheters in a rat model [76], nine in swine/pig models [53,58,60,85,86,87,88,89,90], and one in a mouse model [56]. More inflow catheters showed a higher and more uniform perfusion temperature in rats. *McCabe* et al. was the only study in which flow inversions in a rat model were used, which can be considered as effectively doubling the number of inflow catheters [56]. The average flow velocity in rats was 42 mL per minute, ranging from 10–150 mL per minute. It was not possible to correlate the effect of the flow rate with the intra-abdominal temperature since few studies included thermal measurements (33%). Higher flow rates increase the heat flux into the system of the animal, raising the core temperature. Studies using a flow rate of 10 milliliters per second (n = 4) reported core temperatures ranging from 35–37 °C. All studies using higher flow rates (n = 3) reported core temperatures above 37 °C. These measurements agree with the in silico simulated results found by Löke et al. [95]. As such, the choice of the flow setup can have major consequences on the treatment efficacy.

Elevated temperature can have a profound effect on the efficacy of the chemotherapy used. Therefore, if no thermal homogeneity is achieved, it will become difficult to fairly assess the effect of different choices in all other research parameters. Thermal measurements are also crucial for assuring thermal homogeneity. One temperature probe in the peritoneal cavity, as is most often used, is not enough to evaluate the thermal distribution. Four thermal probes, not tied to the catheters, are sufficient for treatments in rats. Future experiments could determine the minimal amount of probes required for different animals. Reporting measured core and in- and outflow temperatures should become standard in both experimental and clinical settings, providing much-needed thermal data. Unfortunately, these data are currently generally missing in the HIPEC research community.

### 4.4. Temperature Monitoring and Control

Elevated peritoneal temperatures can result in systemic overheating of the animal. Therefore, it is important to monitor the core temperature during treatment and to prevent overheating by cooling if necessary. Only 23% of the included articles reported the core temperature during HIPEC treatment. In almost all cases the rectal temperature was used as a reference, sometimes the temperature under the tongue was measured. Rectal temperatures varying from 32–39 °C were observed in rats, mice, and pigs, whereas 37.5–38 °C was measured under the tongue of rats. Core temperatures of rabbits during treatment were not reported. Several techniques were employed to heat/cool the core of the animal. Often, animals were placed on a heating mattress that was only heating before and after the HIPEC treatment. Cooling of the tail or body during HIPEC with water or ice was reported in some articles, preventing overheating during HIPEC. In some cases, the animals were placed underneath a heating lamp or a warmth chamber to recover from the treatment.

The heat sink effects vary between differently sized animals, and therefore the heating and cooling procedure should be adapted to animal size. Smaller animals, e.g., mice and rats, cool down fast when under anesthesia, but also heat up fast when starting the HIPEC procedure. For these animals, it is extremely important to carefully monitor the core temperature during the entire surgical procedure and to start heating or cooling in time upon temperature change. In larger animals, the core temperature is more stable, and therefore extensive heating or cooling is usually not required. When core temperature profiles from preclinical studies need to reflect the human clinical situation, larger animals with a representative size to humans should be used, e.g., pigs.

Furthermore, the location where the core temperature is measured is crucial. Currently, no studies are comparing different locations to measure the core temperatures, but rectal temperature measurements are likely not adequate enough since the placement of the probe is close to the area perfused by HIPEC and rectal temperatures, therefore, tend to rise rapidly. Preferably, core temperatures should be monitored further away from the perfused area, e.g., underneath the tongue or ideally in the esophagus. The distance to the heated area is then significant and, in this way, it is possible to deduce whether the brain of the animal may get overheated. To further investigate the effect of elevated temperature on the systemic level, it is necessary that reporting the core temperature profile during HIPEC becomes standard.

## 5. Physiological and Anticancer Aspects of Preclinical HIPEC Models

Below we discuss the HIPEC parameters that are relevant to investigate during preclinical HIPEC studies. Depending on the goal and the parameter tested, the desired model can be described as a physiological or anticancer model. This differentiation is relevant for the choices in model components described in the previous section.

### 5.1. Type of Drug and Drug Concentration

Drugs applied during HIPEC are preferably non-cell cycle specific and synergistic with heat as the treatment duration is relatively short and elevated temperatures are used. Platinum-based drugs display a temperature-dependent synergy with heat, resulting in more drug uptake, leading to DNA damage and apoptosis at elevated temperatures in human colorectal cancer cells [22,23]. Overall, the most often used drugs applied during HIPEC in patients are mitomycin C and platinum-based drugs, including oxaliplatin, cisplatin, and carboplatin [14].

The most often used drugs in the reviewed preclinical animal studies were: mitomycin C, cisplatin, oxaliplatin, paclitaxel, and doxorubicin. In Table 3 the frequency and dosage range applied in the evaluated studies are described. Note that some studies applied different types of drugs during the treatment. Studies using colorectal cancer as a tumor model most frequently used oxaliplatin and mitomycin C, whereas cisplatin was only applied in rats with PM originating from ovarian cancer. Cisplatin, mitomycin C, doxorubicin, docetaxel, and carboplatin were applied in PM animal models originating from gastric cancer. The applied temperature shows a wide range for all used drugs with temperatures between 39–44 °C.

The ideal type of drug for HIPEC can be determined in preclinical models. The anticancer activity involving the effect of hyperthermia on chemotherapy can be best investigated in mice or rats in which small tumor lesions throughout the abdominal cavity can be established and a homogeneous temperature distribution can be ensured. Syngeneic animal models provide a representative microenvironment and are therefore suitable for applying immunotherapy or studying the immune response. Research focusing on drug sensitivity or tumor biology requires a tumor model representing the histological and molecular properties of the originating clinical material obtained from patients. Studying the tumor penetration of the drug can be performed in all tumor models.

### 5.2. Carrier Solution

One should be aware of the possible physiological effects of the choice of carrier solution. The synergy between the chemotherapy and the carrier solution can influence the treatment efficacy. For example, dextrose solutions used to be the preferred choice when administering oxaliplatin. The main argument underlying this choice is that oxaliplatin becomes unstable in saline-based carrier solutions, with degradation of around 10% after just 30 min [96], the general duration of an oxaliplatin-based HIPEC treatment. However, the degradation is not linear and after 2 h, 80% of the oxaliplatin is still intact. Furthermore, the degradation product is [Pt(dach)Cl2], which is the active form of the drug [96]. Exposure to dextrose solutions can have adverse effects since hyperglycemia can occur in up to 86% of patients [97]. The underlying metabolic and electrolyte disturbances also cause hyponatremia and hyperlactatemia. The consequence is an increased risk of morbidity [98]. The profound effect of the type of carrier solution on the efficacy of oxaliplatin-based HIPEC treatments makes it an interesting and relevant subject for further in vivo research.

The type of carrier solution also plays a key role in the pharmacokinetics of HIPEC. An interesting study performed by Park et al., providing a striking example of the physiological effect of carrier solution, combined oxaliplatin or mitomycin C with either 1.5% Dianeal peritoneal dialysis solution, 5% dextrose solution, or 20% lipid solution [43]. Drug concentrations in the peritoneum did not differ between carrier solutions. However, plasma concentrations did vary significantly. For mitomycin C the AUC ratio between peritoneum and plasma was 3 times higher for the lipid carrier solution compared to the Dianeal carrier solution. For oxaliplatin, plasma concentrations for lipid and Dianeal were similar, but oxaliplatin carried by dextrose resulted in a significantly higher plasma concentration, resulting in a possible increase of systemic toxicity. This can be explained by the fact that hydrophobic lipid particles are less likely to pass the plasma membrane of endothelial cells [43]. Another explanation is the osmotic force generated by the hypotonicity of the iso-osmotic 5% dextrose solution, possibly enhancing the penetration depth.

Tonicity of the carrier solution together with the molecular weight/size of the chemotherapy determines the way the chemotherapy diffuses into the tissue. Smaller solutes diffuse more freely and can cross more boundaries like endothelial layers, capillaries, cell membranes, etc. Hypotonicity enhances the movement of the solute but increases systemic exposure. Hypertonicity increases the tolerable treatment duration but limits the drug penetration. Isotonicity seems to be the middle ground, limiting adverse effects but also prohibiting potential benefits [43]. Varying the type of carrier solution, the tonicity, and the type of chemotherapy permits the determination of the optimal carrier solution for the entire chemotherapy spectrum used for HIPEC, which can have important consequences for the AUC ratio and post-surgery morbidity.

### 5.3. Volume

The perfusate volume can be an absolute volume or based on the body surface area (BSA). The choice of either an absolute volume or a BSA-based volume can have an impact on both the physiology and anticancer activity as it can influence the treatment chemotherapy concentration for both the healthy and tumorous tissues. An absolute volume was applied in the vast majority of the studies (63% vs. 7% based on BSA). As presented in Figure 2, the absolute perfusate volume range differs for rats, mice, pigs, and rabbits with 27–500 mL, 2–100 mL, 1–10 L, and 250 mL–1 L, respectively. The volume used in pigs is most comparable with the clinical setting where 2 L is an often used absolute perfusate volume.

The volume-based on BSA with 2 L/m^2^ was applied in both rats and pigs. In mice, 4 and 6 L/m^2^ were applied as well. The BSA-based volume does resemble the peritoneal cavity volume and results in a more stable drug concentration compared to absolute perfusate volume. Lemoine et al. studied the effect of BSA-based (150 mg/m^2^) and concentration-based (75 mg/L) HIPEC with oxaliplatin in rats. Although the platinum concentration in the peritoneal tumors was higher for the concentration-based group, median overall survival did not differ between the treatment groups [54].

In mice, the impact of perfusate concentration was assessed by treating the animals with different perfusate volumes (2, 4, or 6 L/m^2^) and with a fixed oxaliplatin concentration of 460 mg/m^2^ [55]. Decreasing the perfusate volume resulted in more toxicity, morbidity, and mortality. Therefore, Liesenfeld et al. recommended that the dosage should be adjusted to the volume to achieve a consistent drug concentration and to standardize the drug concentrations to minimize toxicity and optimize anticancer activity.

### 5.4. Temperature

Increased temperature is one of the key elements of a HIPEC treatment. Heat during HIPEC showed a beneficial effect on the 5-year survival [99]. The cytotoxicity of the chemotherapy used is enhanced by a temperature-dependent factor, the thermal enhancement ratio (TER). In general, three hyperthermic ranges can be distinguished: mild (39–41 °C), moderate (41–43 °C) and severe (>43 °C) hyperthermia [14]. Severe hyperthermia can cause harm to healthy tissues and is not used during HIPEC in clinical practice. Mild and moderate hyperthermia both increase the blood flow to the tissues, stimulate the immune response and increase the cytotoxicity of the chemotherapy in a temperature-dependent way [100,101]. In the evaluated studies, moderate hyperthermia is the most frequently used type of hyperthermia (71% versus 29% mild hyperthermia).

The effect on anticancer activity can be presented by an enhancement curve constructed from TER values obtained at various temperatures, which is dependent on the type of chemotherapy and cell line. The beneficial effects of hyperthermia on a molecular and macroscopic level have been thoroughly described [102,103,104]. On a molecular level, hyperthermia can result in direct cell killing and inhibits DNA damage repair. Aside from the molecular effects, there are several effects on a macroscopic scale that are associated with hyperthermia. These effects include increased perfusion, higher cell permeability, reoxygenation, and alterations to the micro- and macroscopic environment that make tissues more susceptible to chemotherapy [105,106]. In general, the molecular effects can be increased by elevated temperatures, whereas macroscopic effects can be limited. For example, perfusion is decreased during severe hyperthermia, and therefore, perfusion is maximized at a certain hyperthermic temperature. The combination of both effects culminates in a clinical thermal enhancement curve which can vary per type of chemotherapy and cell line.

**Figure 4:**
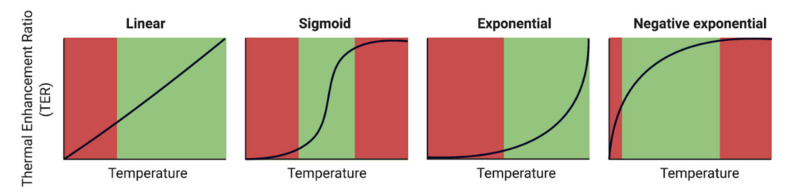
Possible enhancement curves, varying per chemotherapy/cell line combination. The green shaded areas depict the region in the thermal enhancement where the increasing temperature might yield a significantly more effective treatment. The red region on the right visualizes regions where adverse effects such as thermal damage do not outweigh the added thermal enhancement, whereas the left red region visualizes regions where no increased effect or damage is expected. Note that these are simplified curves and that behavior is strongly dependent on the chemotherapy agent and cell line. We assumed that the amount of thermal damage is linear with temperature.

The possible enhancement curves can be classified as either exponential, sigmoid, negative exponential, or linear. Note that these are simplified representations of true enhancement curves for chemotherapeutics, but they are still useful to characterize the general behavior that can be encountered. Linear and exponential curves are observed to have both micro- and macroscopic effects. Sigmoid or negative enhancement curves flatten at a certain temperature and are therefore determined only by macroscopic hyperthermic effects. When chemotherapy/cell line combinations result in an exponential thermal enhancement curve, it becomes more relevant to maximize the HIPEC temperature than for chemotherapy/cell line combinations that produce a sigmoid or negative exponential curve, where temperature differences do not make a significant difference after reaching a certain temperature elevation. Different possible enhancement curves are shown in Figure 4. In this figure, we assumed that the thermal damage to the healthy tissue is linear with temperature such that small increases in enhancement do not outweigh the linear increase in thermal damage to the healthy tissue, as is depicted by the red shaded areas.

For all chemotherapy/cell line combinations, such an enhancement curve can be created, from which the ideal treatment temperature can be determined. During in vitro studies, a first selection can be made for the chemotherapy/cell line combinations, in which the beneficial effects of heat can be determined. The ideal temperature to induce the maximum amount of cell kill in combination with a tolerable level of normal tissue toxicity should be determined during in vivo studies. To determine the optimal temperature, both microscopic and macroscopic effects combining cell line and chemotherapeutics preselected during in vitro studies should be incorporated. If the focus is on cell death and/or penetration, small animals such as mice and rats are recommended. To investigate the physiological effect of the temperature, larger animals are needed to correctly represent human clinical settings. If the goal of the study is to compare chemotherapeutics, one should consider administering them at their own respective ideal temperatures to make a fair, clinically relevant comparison. Preselection during in vitro experiments could determine the optimal temperature range.

### 5.5. Duration

The decision on the duration of HIPEC treatment should consider several factors. The first is the thermal tolerance of the patient, which can be considered as a physiological model. In humans, regimens of 43 °C are possible for a short duration, e.g., 30 min, without causing unwanted systemic effects. For small animals, the heat applied during a HIPEC results more easily in systemic overheating with the risk of significant thermal damage or even death. The total heat applied to the subject can be seen as the time integral over the heat carried by the fluid flow, depending on the volumetric flow rate.

The second consideration regarding the choice of duration is the effect of chemotherapy on anticancer activity. The absorption rate, AUC ratio, and the corresponding discharge of chemotherapy to the systemic compartment are factors that can limit the treatment duration. Oxaliplatin is known as a drug with a relatively low AUC ratio, i.e., the ratio between the peritoneal AUC and plasma AUC, and with a high absorption rate. Therefore, oxaliplatin is often given in high doses for a short duration. Studies that used a very short treatment duration often use oxaliplatin as their preferred chemotherapy. Instead, some studies used oxaliplatin for similarly long durations as studies using mitomycin C, 5-FU, or other chemotherapeutics.

Data from in vitro studies suggest that there exists an optimal treatment duration in which hyperthermia and chemotherapy have a maximal effect. For example, Kirstein et al. showed that the combination of heat (42 °C) and oxaliplatin was significantly more effective at a treatment duration of 2 h compared to 30 min [11]. This was underlined by the study performed by Löffler et al., concluding that 30-min exposure to clinical oxaliplatin concentrations frequently fails to induce 50% cell death and that oxaliplatin should be applied longer for generating an adequate amount of cell death [107]. In a different study by Murata et al. three different gastric cancer cell lines were treated with either 5-FU, mitomycin C or cisplatin for a duration of 30 or 60 min. They concluded that under hyperthermic conditions, growth-inhibitory effects were similar between the two treatment duration for most cell line and chemotherapy combinations. Longer treatment duration showed higher effectiveness for the MKN7 and MKN45 cell lines treated with cisplatin and GCIY cell lines treated with mitomycin C, underlining the cell-line dependent effect of the chemotherapeutics [24]. These studies strongly suggest that duration is an important parameter, which greatly affects the efficacy of the drug.

Francescutti et al. varied the treatment duration for intraperitoneal lavage of low-dose (6 µg/mL) and high-dose (8 µg/mg) mitomycin C, corresponding to a total dose of 15 µg and 20 µg, respectively [49]. The BALB/c mice were treated for either 60 min or 90 min at normothermic temperatures. Using magnetic resonance imaging, tumor volumes were evaluated after 19 days after intraperitoneal chemotherapy. High doses and long treatment duration resulted in, on average, lower tumor volume compared to the combinations high dose/short duration, low dose/short duration, and low dose/long duration. In this study, the survival of mice was determined as well. High dose and long duration improved the survival of the mice (*p* < 0.05). However, there are some concerns regarding this study. The fluid was not heated, while this is known to have a significant effect on the treatment outcome [99], and the applied lavage was not flushed through the abdomen as is standard during clinical HIPEC treatments. The intraperitoneal chemotherapy was injected using 2.5 mL saline as a carrier solution. The solution was distributed using a programmable shaker. These clear drawbacks do not undermine the conclusion that treatment duration can significantly impact the treatment outcome. Future studies should apply hyperthermia and continually flush the fluid through the abdomen to accurately mimic clinical HIPEC conditions.

### 5.6. Delivery Technique

Most evaluated studies applied HIPEC via the open technique (57%), followed by closed (36%) and other techniques (7%). Other delivery techniques used were laparoscopic or semi-open. The design of the semi-open delivery technique was different per study. Löke et al. performed semi-open HIPEC in rats by temporarily closing the abdomen during the procedure using a container construction that effectively separates the outflow region from the peritoneal cavity [76]. The semi-open technique performed in pigs used a covered abdominal cavity expander [84].

Almost all evaluated studies featured only one delivery technique, but five studies compared different delivery techniques. McCabe et al. performed HIPEC in mice using both the open and closed delivery techniques to provide technical guidelines [56]. Less intraoperative complications were experienced using the closed delivery technique, however only the surgical procedure was performed. The effect of the delivery technique on the temperature or drug distribution was not assessed. Badrudin et al. treated eleven rats with pemetrexed for 25 min at 40 °C using either the open or closed delivery techniques [57]. Peritoneal tissue concentration showed no difference, but higher systemic concentrations were observed using the open delivery technique, suggesting it could increase systemic toxicity.

Also, pigs treated with oxaliplatin at 42–43 °C for 30 min using the open delivery technique showed higher systemic absorption and abdominal tissue penetration of oxaliplatin, compared to the open setup [60]. Sánchez-García et al. showed a more constant and homogeneous temperature and drug distribution when pigs received HIPEC via closed HIPEC supported by CO_2_ infusion increasing the distribution throughout the peritoneum [89]. Besides the open and closed techniques, also laparoscopic HIPEC may be safe and feasible in pigs. Peritoneal drug distributions were not significantly different and less systemic uptake of paclitaxel was observed compared to the closed or open HIPEC technique [59].

The optimal delivery technique in clinical situations can be best determined by employing physiological models. Study results obtained in pigs are very relevant since the size and anatomy are similar to the human situation. The delivery technique has an impact on the temperature and drug distribution, resulting in an increased or decreased anticancer activity, but also with the chance of increased systemic toxicity. Each delivery technique has its own advantages which should be taken into account. Closed delivery results in higher tissue penetration, but surgical manipulation to optimize homogeneity is not possible. Furthermore, the surgeon loses access to the peritoneal surface if bleeding occurs during HIPEC. The open technique does provide access during HIPEC, but heat loss from the opened abdomen can lower the overall treatment temperature.

## 6. Outlook

HIPEC treatment protocols should be optimized by investigating all components of the HIPEC treatment. Preclinical research can contribute to a more objective and scientific foundation of HIPEC treatment protocols. Positive outcomes during animal studies can be very helpful to design clinical (feasibility) studies to further optimize clinical treatments. For example, when a parametrical change shows increased survival in an animal study, this can indicate that it might be worthwhile to design a clinical study to test a similar parametrical change in a clinical setting. On the other hand, when the outcome shift due to a parametrical change is relatively small, can be considered trivial, or sufficiently substantiated during preclinical research, a follow-up in a clinical setting is deemed unnecessary. The clinical interpretation of animal study results is strongly dependent on model and HIPEC parameters used and therefore careful study design parameter selection is very important. Even with those precautions, preclinical tumor models have limitations in how well they can represent the response in clinical application in humans. Up to now, substantial preclinical HIPEC research has been performed providing important and clinically relevant insights. In this article, we broke down HIPEC research into several research categories that can be translated to clinically relevant treatment parameters. Below a research framework is outlined that could help to improve future preclinical HIPEC research.

First and foremost, the delivery of HIPEC, specifically the thermal and drug distributions, should be well-controlled. If there are unknown large temperature variations in treatment delivery in and between animals, it is impossible to make a fair comparison between different choices composing the HIPEC treatment. Flow optimization should be performed for experimental setups to reduce these variations. Variations will persist, but these variations have to be quantified such that they can be taken into account while analyzing experimental results.

**Figure 5:**
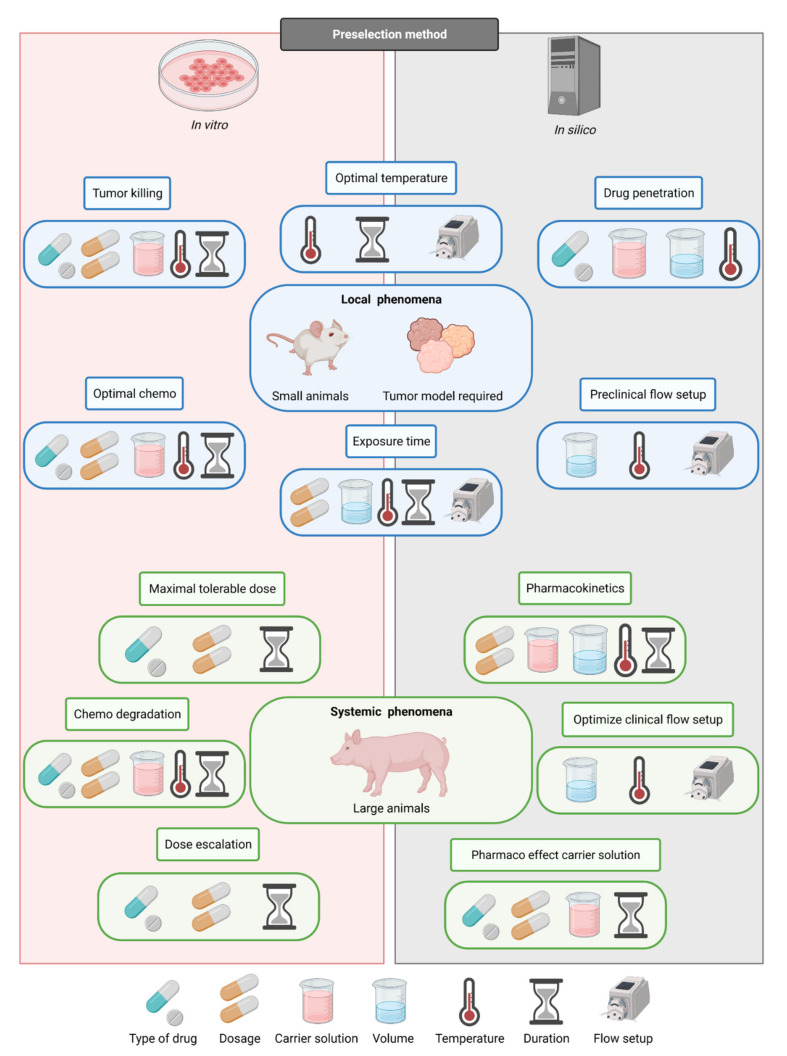
Recommendations for determination of treatment parameters.

When the control and uniformity of the treatment can be ensured, other parameters can be compared. There are numerous cell line and chemotherapy concentration combinations. For example, there are 9 chemotherapeutics widely used for HIPEC. There are 5 tumor types relevant for experimentation based on the PM origins; colorectal, gastric, ovarian, pseudomyxoma peritonei (PMP), and malignant pleural mesothelioma (MPM). Duration can vary between 30 min and 120 min. The chemotherapeutics all interact differently with the carrier solution. Combining all these variables would require the use of an enormous amount of lab animals. Therefore, preselections should be made to reduce the number of experiments needed to obtain the optimal choices. In silico studies can be used to investigate the influence of the setup on the conditions inside the peritoneal cavity. In vitro research can preselect the chemotherapy/cell line combinations, estimate the minimal duration needed for the chemotherapy to work, and exclude thermal ranges and combinations of chemotherapy and carrier solutions. In the left panel of Figure 5, our recommendation to determine the optimal choice of each parameter is defined. For each study goal, the important treatment variables are indicated and which type of animal should be used during in vivo experiments, also indicating whether a tumor model is required, depending on the physiological or anticancer nature of the model. On the left side of the figure, it is indicated if certain study goals need in vitro or in silico preselection of HIPEC research categories. It is very important to decide on the correct order in which the parameters should be determined since most parameters are cross-dependent, as is visible in Figure 5. However, it is important to consider the cross-dependability during the design, experimentation and especially, during the analysis. This way, future preclinical studies provide strong guidance toward optimal HIPEC protocols, which can subsequently be tested in a clinical setting. This trajectory is a faster and safer approach to provide optimal HIPEC treatments and improve clinical outcomes.

## 7. Conclusions

This review provides an overview of in vivo HIPEC methods in animal models and their clinical relevance. Various choices making up the preclinical model, treatment parameters, and flow setup and their consequences were presented and discussed.

Several treatment parameters were evaluated, aiming at providing insights relevant for clinical translation. It is concluded that further research is still needed for solid scientific evidence. The choice of animal type and the presence of an established tumor line are crucial to consider for designing a successful preclinical HIPEC model. We distinguished two different treatment scales relevant for investigating systemic and local phenomena. To increase the translatability we recommended that large animals such as pigs should be used to investigate the systemic phenomena. The establishment of PM is necessary for local phenomena such as anticancer activity and therefore, small animals are recommended. Depending on the research questions, several choices can be made regarding the treatment parameters. Recommendations and possible pitfalls on the choice of type of animal and tumor model per stratified parameter and study goal are provided in this review. The guidelines presented in this paper can improve the clinical relevance, translation, and impact of future in vivo HIPEC experiments.

## Figures and Tables

**Table 1:** Research categories with the corresponding number and study goals of the included HIPEC research papers. Recommendations on the type of animal and tumor model for future research.

Research Category Effect of:	Number of Papers	Reference(s)	Study Goal	Recommended Animal Model	Recommended Tumor Model
1. Type of drug	15	[32,33,34,35,36,37,38,39,40,41,42,43,44,45,46]	Uptake and/or sensitivity of tumor tissue	Mouse, rat	Syngeneic, xenograft or PDX
			Immune response	Mouse, rat	Syngeneic
2. Drug concentration	11	[32,36,40,47,48,49,50,51,52,53,54]	Uptake by ‘healthy’ organs	Pig	Not required
			Uptake and/or sensitivity of tumor tissue	Mouse, rat	Syngeneic, xenograft or PDX
3. Carrier solution	1	[43]	Drug effectiveness	Mouse, rat	Syngeneic, xenograft or PDX
			Systemic toxicity	Pig	Not required
4. Volume	1	[55]	Drug and temperature distribution	Pig	Not required
			Drug effectiveness	Mouse, rat	Syngeneic, xenograft or PDX
5. Temperature	2	[51,52]	Effect on drug uptake and/or tumor sensitivity	Mouse, rat	Syngeneic, xenograft or PDX
			Systemic toxicity	Pig	Not required
6. Duration	1	[55]	Uptake and/or sensitivity of tumor tissue	Mouse, rat	Syngeneic, xenograft or PDX
			Systemic toxicity	Pig	Not required
7. Delivery technique	6	[56,57,58,59,60,61]	Drug and temperature distribution in the peritoneal area	Pig	Not required
			Systemic toxicity	Pig	Not required

**Table 2:** Detailed information of the tumor models used in the analyzed studies.

Animal Type	Tumor Model	Cell Line	Cell Amount	Dissolvent	Tumor Take Rate (%)	Tumor Outgrowth Time (Days after Injection)	PCI Score	Location(s)	Ref.(s)
Rats	Syngeneic	CC531	2 × 10^6^	PBS	100	7–8	6–10	Greater omentum, liver hilum, perisplenic area, mesentery, bowel surface, gonadal fat pads, intra-abdominal site of inoculation	[47,54,69,70,72,73,74]
Rats	Syngeneic	Ovarian cancer cells	1 × 10^7^	Ascitic liquid + saline (1:4)	64–100	-	-	Ascites, visceral and parietal peritoneum, greater and lesser omentum, mesentery	[32,45,64]
Rats	PDX	PMCA-3	500 µL ascites	Mucinous tumor tissue		19–24	11	Larger omentum, splenic surface and splenic hilum, liver surface and liver hilum, gonadal fat pads, and parietal peritoneum	[77]
Rats	Syngeneic	DHD/K12 /Trb	2 × 10^5^	-	98	21	18	-	[44]
Rats	Xenograft	SKOV-3	5 × 5 × 3 mm	Not dissolved	100	21	-	Only at the transplantation site	[78]
Mice	Xenograft	OVCAR-3	6.0 × 10^6^	Serum-free DMEM	100	19	-	-	[81]
Mice	Syngeneic	ID8-luc	1 × 10^6^	-	-	5	-	-	[33]
Mice	Xenograft	MKN45	1 × 10^7^	Serum-free medium	100	10	-	Mesentery, diffuse colonization of the peritoneal cavity	[34]
Mice	Xenograft	HCT116	2 × 10^6^ or 2.5 × 10^7^	Not reported or PBS + 500 μg/mL matrigel	100	7–10	20	Small nodules diffused in the peritoneum, mesentery	[50,55]
Mice	Syngeneic	EAT	2 × 10^6^	Saline	-	-	-	Ascites	[36,79]
Mice	Syngeneic	MCA	5 × 10^3^	-	-	-	-	-	[35]
Mice	Xenograft	SHIN-3	5 × 10^6^	PBS	100	27	2–11	Pancreas, peritoneum, liver, small intestine, spleen, ascites, colon, stomach diaphragm	[48]
Mice	Syngeneic	CT26	3 × 10^6^ or 5 × 10^4^	PBS or saline	100	5	-	Small bowel serosa, small bowel mesentery	[49,56]
Mice	Syngeneic	Colon 26	5 × 10^4^	Saline	100	7	-	Mesentery	[82]
Mice	Xenograft	A2780/CP70	1 × 10^6^	Serum-free RPMI 1640	-	21	-	Small bowel, colon	[37]
Mice	Syngeneic	B16F10	1 × 10^6^	-	100	10	9	Small bowel, liver	[38]
Mice	Xenograft	SKOV-3	5.0 × 10^5^	Matrigel	-	14	-	Only at the transplantation site	[80]
Mice	Syngeneic	MC38	2 × 10^6^	-	-	2	-	Perisplenic, peripancreatic, omental fat	[39]
Rabbits	Syngeneic	VX2	5 × 10^10^	-	100	8	9.5	Greater omentum, antrum of the stomach, abdominal wall, diaphragm, intestinal wall	[41,42]

PCI: Peritoneal Cancer Index; PDX: Patient-Derived Xenograft.

**Table 3:** Overview of the applied drugs in percentages with the corresponding dosage range and applied temperature range.

Type of Drug	Rat (%; Dosage)	Mouse (%; Dosage)	Pig (%; Dosage)	Rabbit (%; Dosage)	Temperature Range (°C)	Clinical Dosage (mg/m^2^) [14]
Mitomycin C	36%; 1.5–4 mg or 2 mg/kg or 15–35 mg/m^2^	25%; 6–8.25 µg/mL or 5 mg/kg	-	-	40–44	10–160
Oxaliplatin	21%; 77.5 mg/kg or 150–1840 mg/m^2^	13%; 460–920 mg/m^2^	38%; 400 mg or 150 mg/mL or 360–460 mg/m^2^	-	40–43	160–460
Paclitaxel	14%; 0.24 mg/mL or 60 mg/m^2^	-	23%; 175 mg/m^2^	33%; 10.83 mg/kg	40–43	60–175
Cisplatin	7%; 4–40 mg/kg	50%; 3–37.5 mg/kg or 70–75 mg/m^2^	15%; 70 mg/m^2^	-	39–43.5	50–360
Doxorubicin	7%; 2 mg/kg	6%; not reported	-	33%; not reported	40–43	15
Other	18%	25%	-	33%	40.5–43	-

## Supplementary Materials

The following are available online at https://www.mdpi.com/article/10.3390/cancers13143430/s1, Table S1: Papers included in this study with the parameters relevant for the efficacy of HIPEC.
