# Supplementary material for: Preclinical In Vivo-Models to Investigate HIPEC; Current Methodologies and Challenges
Source: Cancers (Basel). 2021 Jul 8;13(14):3430. doi: 10.3390/cancers13143430 (PMC8303745; doi:10.3390/cancers13143430)
Supplement: Supplementary file 1 [file cancers-13-03430-s001.zip › cancers-1276743-supplementary.pdf]

Supplementary Table 1. Papers included in this study with the parameters relevant for the efficacy of HIPEC.

Animal	PM cells	Delivery	In-flows	Out-flows	Flow rate	Duration (min)	Volume	Carrier solution	Drugs	Dose	Temperature (°C)	Temperature reported (Y/N)	Core	Cooling/ Heating	Flushing	Type of animal	Reference
Rats	None	Open	1	1	100-150 ml/min	30	-	NaCl 0.9%	Paclitaxel	60 mg/m²	43	N	-	-	N	Sprague–Dawley	[1]
Rats	None	Open	1	1	10-15 ml/min	90	500 ml	Saline	MMC	20 mg/m²	40.5-41	N	-	-	N	Pathogen-free Wag	[2]
Rats	None	Semi-open	1 or 4	1	1.6 ml/s	90	500 ml	PBS	None		41.5-42.3	Y	37.5-38	Ethanol on tail	N	Athymic nude	[3]
Rats	None	Closed	1	1	-	90	300 ml	Saline	Cisplatin	5 mg/kg	42	N	-	-	N	Wistar-albino	[4]
Rats	None	Open	1	1	30 ml/min	45	100 ml	PBS + 0.1% HPMC	Paclitaxel (RAME-b-CD)	0.24 mg/ml	41	N	-	-	N	Wag/Rij	[5]
Rats	None	Open	1	1	30-40 ml/min	60 (Saline), 90 (MMC, Cis, Doxo)	300 ml	NaCl 0.9%	MMC, Cisplatin, Oxaliplatin, Doxorubicin	2 mg/kg, 5 mg/kg, 25 mg/kg, 2 mg/kg	42	N	-	-	N	Wistar-albino	[6]
Rats	CC531	Open	1	1	10 ml/min	30	250 & 2 L/m2	NaCl 0.9%	Oxaliplatin	150 mg/m²	40.2	Y	35	Heating pad	N	Wag/Rij	[7]
Rats	None	Open	1	1		60	-	Saline	Oxaliplatin	77.5 mg/kg	42	N		-	N	Wistar-albino	[8]
Rats	Ovarian cancer cells	-	1	1	15 ml/min	45	-	Saline	Diodaxet	15 mg/kg	40.9	Y	38.5	-	Y	Wistar	[9]
Rats	Ovarian cancer cells	Open	1	1	10-15 ml/min	45	200 ml	NaCl 0.9%	Cisplatin or Dioxaded	20 or 15 mg/kg	40.5-41.5	N	-	Heating table	Y	Wistar	[10]
Rats	CC531	Open	1	1	30 ml/min	45	-	PBS + 0.1% HPMC	Paclitaxel	0.24 mg/ml	41	N	-	-	N	Wag/Rij	[11]
Rats	PMCA-3	Open	1	1	40 ml/min	90	150 ml	NaCl 0.9%	MMC	1.5 mg	41.7	Y	-	Ice and water	N	Rowett nude	[12]

Rats	Colonic adeno-carcinoma cells DHD/K12 /Trb	Open	1	1	60 ml/min	60	250 ml	Saline	MMC or Gemcitabine	15 mg/m² or 24 mg/kg	41.2-42.3	Y	-	-	N	BD IX/HansHsd	[13]
Rats	None	Open	1	1	40 ml/min	60	300 ml	Dianeal vs Dextroxe 5% vs Lipision	MMC or Oxaliplatin	35 or 460 mg/m²	41-42	N	-	-	N	Sprague–Dawley	[14]
Rats	CC531	Closed	1	1	-	60	250 ml	NaCl 0.9%	MMC	4 mg	44	N	-	-	Y	WAG/Rij	[15]
Rats	Ovarian cancer cells	-	1	1	-	-	-	Saline	Dioxaded or Cisplatin	1.5, 15, 30 or 4, 20, 40 mg/kg	40.5-41.5	N	-	-	N	Wistar	[16]
Rats	CC531	Open	1	1	-	90	250 ml	Saline	MMC	15 mg/m²	40.5-41.5	Y	37.8	-	Y	Pathogen-free WAG/Rij	[17]
Rats	CC531	Closed	1	1	10 ml/min	90	250 ml	NaCl 0.9%	MMC	15 mg/m² & 35 mg/m²	42	Y	36	Warmed mattrass	Y	WAG/Rij	[18]
Rats	CC531	Closed	1	1	10 ml/min	90	250 ml	NaCl 0.9%	MMC	35 mg/m²	41.9	Y	37	Warmed mattrass	Y	WAG/Rij	[19]
Rats	None	Open	1	1	80 ml/min	90	150 ml	NaCl 0.9%	Melphalan	12 mg/kg	42	Y	38	-	N	Sprague–Dawley	[20]
Pigs	None	Closed	1	1	-	30	6-10 L	50 g/L glucose	Oxaliplatin	150 mg/L	42	N	-	-	Y	White	[21]
Rats	None	Closed	1	1	100 ml/min	60	300 ml	5% dextrose	Doxorubicin	2 mg/kg	41-43	Y	-	-	N	Sprague–Dawley	[22]
Rats	SKOV-3	Open	1	1	-	45	125 ml	NaCl 0.9%	Paclitaxel	0.24 mg/ml	37	N	-	-	N	Athymic nude	[23]
Rats	CC531	Open	1	1	10 ml/min	90	250 ml	NaCl 0.9%	MMC	35 mg/m²	41.3	Y	37	Warmed mattrass	N	Wag/Rij	[24]

Pigs	None	Open, internal heating	1	1	-	60	3 L	Physiologic serum	None	None	42	N	39	-	N	White	[25]
Pigs	None	Closed	2	1	30 ml/min	60	2 L	Isotonic saline + ringer	Cisplatin	70 mg/m^2	43	N	-	-	N	Sus scrofa domesticus	[26]
Mice	OVCAR-3	Closed	1	1	0.08 ml/s	12	100 ml	Saline	Oxaliplatin	920 mg/m^2	43	Y	32-39	Warmed mattress	N	C57BL/6	[27]
Mice	ID8-luc	-	1	1	6 ml every 20-30 seconds	20	6 ml	PBS	Paclitaxel & Cisplatin	10 mg/kg 6 mg/kg	41-43	N	-	Heater light	N	C57BL/6	[28]
Mice	MKN45	Open	1	1	4 ml/min	50	-	saline	MMC & Cisplatin	8.25 & 62.5 ug/L	40	N	-	Heated cage after treatment	Y	NOD-SCID	[29]
Mice	HCT-116 & HT-29	-	1	1	300 ml/h	30 & 90	4 or 6 L/m^2	5% glucose & Nacl 0.9%	Oxaliplatin	460 mg/m^2	42	N	-	Cooling by bolus	N	MozygousCrl: NU [Ncr]-Foxn1n	[30]
Mice	EAT cells	Closed	-	-	-	-	2x2 ml	NaCl 0.9%	Cisplatin	5-10 mg/kg	42.5-43	Y	-	-	N	Swiss albino	[31]
Mice	MCA	Closed	-	-	-	35	2x2 ml	Saline	Doxorubicin, Cisplatin, MMC, 5-FU	20 mg/kg, 10 mg/kg, 5 mg/kg, 150 mg/kg	38.2-43.5	Y	36-38	-	N	CBA inbred	[32]
Mice	SHIN-3	Open	1	1	3 ml/min	60	-	-	Cisplatin	37.5 mg/ml or 75 mg/m^2	39	N	-	Heat pad	N	NMRI-nu (nu/nu)	[33]
Mice	CT26	Open or closed	1	1 or 2	200 ml/h or 50 ml/h (open or closed)	30	-	NaCl 0.9%	-	-	-	N	-	Water pad	Y	BALB/c	[34]
Mice	EAT cells	Closed	1	1	-	-	2x2 ml	NaCl 0.9%	Cisplatin, quercetin	5 or 10 mg/kg and 50 mg/kg	43	N	-	-	N	Swiss albino	[35]

Mice	Colon 26	Closed	1	1	2.5 ml/min	10-30	-	Saline	-	-	43-44	Y	< 37	Warmth chamber after treatment	N	BALB/c	[36]
Mice	A2780/CP 70	Closed	1	1	3 ml/min	60	100 ml	Saline	Cisplatin + sodium arsenite + cefazolin	3 mg/kg + 26 mg/kg + 0.01 mg/ml	40-43	Y	36.3	Heating pad + heat lamp	N	NCr athymic nude	[37]
Mice	Colon 26	Open	1	1	-	60-90	2.5 ml	Saline	MMC	6-8 ug/ml		N	-	Heating pad	Y	BALB/c	[38]
Mice	B16F10	Open	1	1	-	30	-	Saline	Dacarbazine & Nivolumab	15 & 10 mg/m^2	42	N	-	Heating lamp	Y	Swiss albino	[39]
Mice	HCT 116-luc	Closed	1	1	-	30	-	Saline	Raltitrexed	3.75-7.5 mg/m^2	43	N	-	-	Y	Nude	[40]
Mice	SKOV-3-Luc IP1	Open	1	1	-	60	-	Saline	Cisplatin	70 mg/m^2	40-41	N	-	-	N	Nude-foxn1nu	[41]
Mice	MC38	Open	1	1	-	-	-	Balanced dialysis fluid	MMC + Doxorubicin		40	N	-	Recovered on heating pad	Y	C57Bl/6	[42]
Rats	None	Open	1	1	27 ml/min	25	27 ml	D5W	Oxaliplatin or MMC	460 or 10 and 35 mg/m^2	43	N	-	-	Y	Sprague–Dawley	[43]
Rats	None	Closed	1	1	12 ml/min	25	100 ml	Saline	Pemetrexed	500, 1000, 1500 mg/m^2	40 & 43	N	-	Heated mattresses	N	Sprague–Dawley	[44]
Rats	None	Open or closed	1	1	12 ml/min	25	100 ml	Saline	Pemetrexed	1000 mg/kg	40	N	-	Heated mattresses	N	Sprague–Dawley	[45]
Rats	None	Closed	1	1	18.75 ml/min	25	67 ml	Saline	Oxaliplatin	460, 920 and 1840 mg/m^2	40 & 43	N	-	-	N	Sprague–Dawley	[46]
Rats	None	Closed	1	1	26 ml/min	25	93 ml	Dextrose	Oxaliplatin	460 mg/kg	40	N	-	Mattress before perfusion	N	Sprague–Dawley	[47]

Pigs	None	Closed	2	2	-	30	1 L	Dye + saline	None		42	N	-	-	N	Not reported	[48]
Pigs	None	Closed	2	2	-	-	-	-	Paclitaxel	175 mg/m^2	42	N	-	-	N	Not reported	[49]
Pigs	None	Open and closed	2	2	2.4 L/min	60	4 L	1.36% glucose and 25 mmol/L bicar-bonate and dye	Paclitaxel	175 mg/m^2	40-43 (closed) 43-33 (open)	Y	-	-	N	White	[50]
Mini-pigs	None	Closed and open	-	-	2.4 L/min	60	-	1.36% glucose and 25 mmol/L bicar-bonate and dye	Paclitaxel	175 mg/m^2	41-42	N	-	Thermal blanket	N	Mini	[51]
Pigs	None	Closed	2	1	-	60	2 L/m^2	Peritoneal dialysis solution	Cisplatin	70 mg/m^2	42-43	N	-	-	N	Sus scrofa domesticus	[52]
Pigs	None	Open	2	2	1360 ml/min	30	2 L/m^2	Dextrose	Oxaliplatin	460 or 360 mg/m^2	41-43	Y	-	-	N	Not reported	[53]
Pigs	None	Laporasco pic	1 or 4	3	1000 ml/min	30	4 L	Dextrose	Oxaliplatin	400 mg	42	N	-	-	Y	Sus scrofa domesticus	[54]
Pigs	None	Open and closed	2	1		30	4 L	Glucose & dye	Oxaliplatin	400 mg/m2	41.5-42.7 (closed) & 42.1-42.6 (Open)	Y	37.9-38.8	-	N	White	[55]
Pigs	None	Laporasco pic	2	2	1500 ml/min	20	-	Saline	-	-	41-42	Y	-	-	N	Not reported	[56]
Pigs	None	Semi-Open	1	1	-	30	4 L	50 g/L glucose	Oxaliplatin	150 mg/L	42	N	-	-	N	White	[57]
Rabbits	None	Open	1	1	-	60	1 L	NaCl 0.9%	Nab-Paclitaxel	10.83 mg/kg	40-42	N	-	-	N	New Zealand White	[58]

Rabbits	VX2 carcinoma	Open	1	1	10 ml/min	30	250 ml	Saline	Docetaxel and Carboplatin	10 mg and 40 mg	41.5-42.5	N	-	-	N	New Zealand White	[59]
Rabbits	VX2 carcinoma	Open	1	1	10 ml/min	30	250 ml	Saline	Peptide Doxorubicin or Doxorubicin	-	-	N	-	-	N	New Zealand White	[60]

References

1. Lopez-Lopez, V.; Lynn, P.B.; Gil, J.; Garcia-Salom, M.; Gil, E.; Gonzalez, A.; Munoz, I.P.; Cascales-Campos, P.A. Effect of Paclitaxel-based Hyperthermic Intraperitoneal Chemotherapy (HIPEC) on colonic anastomosis in a rat model. *Clin Transl Oncol* **2019**, *21*, 505-511, doi:10.1007/s12094-018-1948-7.
2. Pelz, J.O.; Doerfer, J.; Decker, M.; Dimmler, A.; Hohenberger, W.; Meyer, T. Hyperthermic intraperitoneal chemoperfusion (HIPEC) decrease wound strength of colonic anastomosis in a rat model. *Int J Colorectal Dis* **2007**, *22*, 941-947, doi:10.1007/s00384-006-0246-y.
3. Loke, D.R.; Helderman, R.; Sijbrands, J.; Rodermond, H.M.; Tanis, P.J.; Franken, N.A.P.; Oei, A.L.; Kok, H.P.; Crezee, J. A Four-Inflow Construction to Ensure Thermal Stability and Uniformity during Hyperthermic Intraperitoneal Chemotherapy (HIPEC) in Rats. *Cancers (Basel)* **2020**, *12*, doi:10.3390/cancers12123516.
4. Ocak, S.; Buk, O.F.; Genc, B.; Avci, B.; Uzuner, H.O.; Gundogdu, S.B. The effects of platelet-rich-plasma gel application to the colonic anastomosis in hyperthermic intraperitoneal chemotherapy: An experimental rat model. *Int Wound J* **2019**, *16*, 1426-1432, doi:10.1111/iwj.13207.
5. Bouquet, W.; Ceelen, W.; Adriaens, E.; Almeida, A.; Quinten, T.; De Vos, F.; Pattyn, P.; Peeters, M.; Remon, J.P.; Vervaet, C. In vivo toxicity and bioavailability of Taxol and a paclitaxel/beta-cyclodextrin formulation in a rat model during HIPEC. *Ann Surg Oncol* **2010**, *17*, 2510-2517, doi:10.1245/s10434-010-1028-x.
6. Aghayeva, A.; Benlice, C.; Bilgin, I.A.; Atukeren, P.; Dogusoy, G.; Demir, F.; Atasoy, D.; Baca, B. The effects of hyperthermic intraperitoneal chemoperfusion on colonic anastomosis: an experimental study in a rat model. *Tumori* **2017**, *103*, 307-313, doi:10.5301/tj.5000610.
7. Lemoine, L.; Thijssen, E.; Carleer, R.; Cops, J.; Lemmens, V.; Eyken, P.V.; Sugarbaker, P.; der Speeten, K.V. Body surface area-based versus concentration-based intraperitoneal perioperative chemotherapy in a rat model of colorectal peritoneal surface malignancy: pharmacologic guidance towards standardization. *Oncotarget* **2019**, *10*, 1407-1424, doi:10.18632/oncotarget.26667.
8. Buk, O.F.; Ocak, S.; Genc, B.; Avci, B.; Uzuner, H.O. Is platelet-rich plasma improves the anastomotic healing in hyperthermic intraperitoneal chemotherapy with oxaliplatin: an experimental rat study. *Ann Surg Treat Res* **2020**, *98*, 89-95, doi:10.4174/astr.2020.98.2.89.
9. Bespalov, V.G.; Kireeva, G.S.; Belyaeva, O.A.; Senchik, K.Y.; Stukov, A.N.; Maydin, M.A.; Semenov, A.L.; Gafton, G.I.; Guseynov, K.D.; Belyaev, A.M. Experimental study of antitumour activity and effects on leukocyte count of intraperitoneal administration and hyperthermic intraperitoneal chemoperfusion (HIPEC) with dioxadet in a rat model of ovarian cancer. *J Chemother* **2016**, *28*, 203-209, doi:10.1179/1973947815Y.0000000040.
10. Bespalov, V.G.; Kireeva, G.S.; Belyaeva, O.A.; Kalinin, O.E.; Senchik, K.Y.; Stukov, A.N.; Gafton, G.I.; Guseynov, K.D.; Belyaev, A.M. Both heat and new chemotherapeutic drug dioxadet in hyperthermic intraperitoneal chemoperfusion improved survival in rat ovarian cancer model. *J Surg Oncol* **2016**, *113*, 438-442, doi:10.1002/jso.24140.
11. Bouquet, W.; Deleye, S.; Staelens, S.; De Smet, L.; Van Damme, N.; Debergh, I.; Ceelen, W.P.; De Vos, F.; Remon, J.P.; Vervaet, C. Antitumour efficacy of two paclitaxel formulations for hyperthermic intraperitoneal chemotherapy (HIPEC) in an in vivo rat model. *Pharm Res* **2011**, *28*, 1653-1660, doi:10.1007/s11095-011-0401-1.
12. Sorensen, O.; Andersen, A.M.; Larsen, S.G.; Giercksky, K.E.; Flatmark, K. Intraperitoneal mitomycin C improves survival compared to cytoreductive surgery alone in an experimental model of high-grade pseudomyxoma peritonei. *Clin Exp Metastasis* **2019**, *36*, 511-518, doi:10.1007/s10585-019-09991-0.

13. Raue, W.; Kilian, M.; Braumann, C.; Atanassow, V.; Makareinis, A.; Caldenas, S.; Schwenk, W.; Hartmann, J. Multimodal approach for treatment of peritoneal surface malignancies in a tumour-bearing rat model. *Int J Colorectal Dis* **2010**, *25*, 245-250, doi:10.1007/s00384-009-0819-7.
14. Park, E.J.; Ahn, J.; Gwak, S.W.; Park, K.S.; Baik, S.H.; Hwang, S.J. Pharmacologic Properties of the Carrier Solutions for Hyperthermic Intraperitoneal Chemotherapy: Comparative Analyses Between Water and Lipid Carrier Solutions in the Rat Model. *Ann Surg Oncol* **2018**, *25*, 3185-3192, doi:10.1245/s10434-018-6628-x.
15. Aarts, F.; Bleichrodt, R.P.; de Man, B.; Lomme, R.; Boerman, O.C.; Hendriks, T. The effects of adjuvant experimental radioimmunotherapy and hyperthermic intraperitoneal chemotherapy on intestinal and abdominal healing after cytoreductive surgery for peritoneal carcinomatosis in the rat. *Ann Surg Oncol* **2008**, *15*, 3299-3307, doi:10.1245/s10434-008-0070-4.
16. Bespalov, V.G.; Alvovsky, I.K.; Tochilnikov, G.V.; Stukov, A.N.; Vyshinskaya, E.A.; Semenov, A.L.; Vasilyeva, I.N.; Belyaeva, O.A.; Kireeva, G.S.; Senchik, K.Y.; et al. Comparative efficacy evaluation of catheter intraperitoneal chemotherapy, normothermic and hyperthermic chemoperfusion in a rat model of ascitic ovarian cancer. *Int J Hyperthermia* **2018**, *34*, 545-550, doi:10.1080/02656736.2017.1375161.
17. Pelz, J.O.; Doerfer, J.; Hohenberger, W.; Meyer, T. A new survival model for hyperthermic intraperitoneal chemotherapy (HIPEC) in tumor-bearing rats in the treatment of peritoneal carcinomatosis. *BMC Cancer* **2005**, *5*, 56, doi:10.1186/1471-2407-5-56.
18. Klaver, Y.L.; Hendriks, T.; Lomme, R.M.; Rutten, H.J.; Bleichrodt, R.P.; de Hingh, I.H. Intraoperative hyperthermic intraperitoneal chemotherapy after cytoreductive surgery for peritoneal carcinomatosis in an experimental model. *Br J Surg* **2010**, *97*, 1874-1880, doi:10.1002/bjs.7249.
19. Klaver, Y.L.; Hendriks, T.; Lomme, R.M.; Rutten, H.J.; Bleichrodt, R.P.; de Hingh, I.H. Intraoperative versus early postoperative intraperitoneal chemotherapy after cytoreduction for colorectal peritoneal carcinomatosis: an experimental study. *Ann Surg Oncol* **2012**, *19 Suppl 3*, S475-482, doi:10.1245/s10434-011-1984-9.
20. Glehen, O.; Stuart, O.A.; Mohamed, F.; Sugarbaker, P.H. Hyperthermia modifies pharmacokinetics and tissue distribution of intraperitoneal melphalan in a rat model. *Cancer Chemother Pharmacol* **2004**, *54*, 79-84, doi:10.1007/s00280-004-0779-0.
21. Facy, O.; Combier, C.; Poussier, M.; Magnin, G.; Ladoire, S.; Ghiringhelli, F.; Chauffert, B.; Rat, P.; Ortega-Deballon, P. High pressure does not counterbalance the advantages of open techniques over closed techniques during heated intraperitoneal chemotherapy with oxaliplatin. *Surgery* **2015**, *157*, 72-78, doi:10.1016/j.surg.2014.06.006.
22. Jacquet, P.; Averbach, A.; Stuart, O.A.; Chang, D.; Sugarbaker, P.H. Hyperthermic intraperitoneal doxorubicin: pharmacokinetics, metabolism, and tissue distribution in a rat model. *Cancer Chemother Pharmacol* **1998**, *41*, 147-154, doi:10.1007/s002800050721.
23. Colin, P.; De Smet, L.; Vervaet, C.; Remon, J.P.; Ceelen, W.; Van Bocxlaer, J.; Boussery, K.; Vermeulen, A. A model based analysis of IPEC dosing of paclitaxel in rats. *Pharm Res* **2014**, *31*, 2876-2886, doi:10.1007/s11095-014-1384-5.
24. Klaver, Y.L.; Hendriks, T.; Lomme, R.M.; Rutten, H.J.; Bleichrodt, R.P.; de Hingh, I.H. Hyperthermia and intraperitoneal chemotherapy for the treatment of peritoneal carcinomatosis: an experimental study. *Ann Surg* **2011**, *254*, 125-130, doi:10.1097/SLA.0b013e3182197102.
25. Ortega-Deballon, P.; Facy, O.; Magnin, G.; Piard, F.; Chauffert, B.; Rat, P. Using a heating cable within the abdomen to make hyperthermic intraperitoneal chemotherapy easier: feasibility and safety study in a pig model. *Eur J Surg Oncol* **2010**, *36*, 324-328, doi:10.1016/j.ejso.2009.11.010.
26. Davigo, A.; Passot, G.; Vassal, O.; Bost, M.; Tavernier, C.; Decullier, E.; Bakrin, N.; Alyami, M.; Bonnet, J.M.; Louzier, V.; et al. PIPAC versus HIPEC: cisplatin spatial distribution and diffusion in a swine model. *Int J Hyperthermia* **2020**, *37*, 144-150, doi:10.1080/02656736.2019.1704891.
27. Miailhe, G.; Arfi, A.; Mirshahi, M.; Eveno, C.; Pocard, M.; Touboul, C. A new animal model for hyperthermic intraperitoneal chemotherapy (HIPEC) in tumor-bearing mice in the treatment of peritoneal carcinomatosis of ovarian origin. *J Visc Surg* **2018**, *155*, 183-189, doi:10.1016/j.jviscsurg.2017.10.008.
28. Huang, W.C.; Wu, C.C.; Hsu, Y.T.; Chang, C.L. Effect of hyperthermia on improving neutrophil restoration after intraperitoneal chemotherapy. *Int J Hyperthermia* **2019**, *36*, 1255-1263, doi:10.1080/02656736.2019.1699172.
29. Graziosi, L.; Mencarelli, A.; Renga, B.; Santorelli, C.; Cantarella, F.; Bugiantella, W.; Cavazzoni, E.; Donini, A.; Fiorucci, S. Gene expression changes induced by HIPEC in a murine model of gastric cancer. *In Vivo* **2012**, *26*, 39-45.

30. Liesenfeld, L.F.; Hillebrecht, H.C.; Klose, J.; Schmidt, T.; Schneider, M. Impact of Perfusate Concentration on Hyperthermic Intraperitoneal Chemotherapy Efficacy and Toxicity in a Rodent Model. *J Surg Res* **2020**, *253*, 262-271, doi:10.1016/j.jss.2020.03.067.
31. Orsolic, N.; Car, N.; Lisicic, D.; Benkovic, V.; Knezevic, A.H.; Dikic, D.; Petrik, J. Synergism between propolis and hyperthermal intraperitoneal chemotherapy with cisplatin on ehrlich ascites tumor in mice. *J Pharm Sci* **2013**, *102*, 4395-4405, doi:10.1002/jps.23755.
32. Bevanda, M.; Orsolic, N.; Basic, I.; Vukojevic, K.; Benkovic, V.; Horvat Knezevic, A.; Lisicic, D.; Dikic, D.; Kujundzic, M. Prevention of peritoneal carcinomatosis in mice with combination hyperthermal intraperitoneal chemotherapy and IL-2. *Int J Hyperthermia* **2009**, *25*, 132-140, doi:10.1080/02656730802520697.
33. Derrien, A.; Gouard, S.; Maurel, C.; Gaugler, M.H.; Bruchertseifer, F.; Morgenstern, A.; Faivre-Chauvet, A.; Classe, J.M.; Cherel, M. Therapeutic Efficacy of Alpha-RIT Using a (213)Bi-Anti-hCD138 Antibody in a Mouse Model of Ovarian Peritoneal Carcinomatosis. *Front Med (Lausanne)* **2015**, *2*, 88, doi:10.3389/fmed.2015.00088.
34. McCabe-Lankford, E.; Peterson, M.; McCarthy, B.; Brown, A.J.; Terry, B.; Galarza-Paez, L.; Levi-Polyachenko, N. Murine Models of Intraperitoneal Perfusion for Disseminated Colorectal Cancer. *J Surg Res* **2019**, *233*, 310-322, doi:10.1016/j.jss.2018.07.063.
35. Orsolic, N.; Car, N. Quercetin and hyperthermia modulate cisplatin-induced DNA damage in tumor and normal tissues in vivo. *Tumour Biol* **2014**, *35*, 6445-6454, doi:10.1007/s13277-014-1843-y.
36. Kudo, M.; Asao, T.; Hashimoto, S.; Kuwano, H. Closed continuous hyperthermic peritoneal perfusion model in mice with peritoneal dissemination of colon 26. *Int J Hyperthermia* **2004**, *20*, 441-450, doi:10.1080/02656730310001637352.
37. Muenyi, C.S.; States, V.A.; Masters, J.H.; Fan, T.W.; Helm, C.W.; States, J.C. Sodium arsenite and hyperthermia modulate cisplatin-DNA damage responses and enhance platinum accumulation in murine metastatic ovarian cancer xenograft after hyperthermic intraperitoneal chemotherapy (HIPEC). *J Ovarian Res* **2011**, *4*, 9, doi:10.1186/1757-2215-4-9.
38. Francescutti, V.; Rivera, L.; Seshadri, M.; Kim, M.; Haslinger, M.; Camoriano, M.; Attwood, K.; Kane, J.M., 3rd; Skitzki, J.J. The benefit of intraperitoneal chemotherapy for the treatment of colorectal carcinomatosis. *Oncol Rep* **2013**, *30*, 35-42, doi:10.3892/or.2013.2473.
39. Duzgun, O.; Sarici, I.S.; Gokcay, S.; Ates, K.E.; Yilmaz, M.B. Effects of nivolumab in peritoneal carcinamatosis of malign melanoma in mouse model. *Acta Cir Bras* **2017**, *32*, 1006-1012, doi:10.1590/s0102-865020170120000002.
40. Qiu, C.; Li, Y.; Liang, X.; Qi, Y.; Chen, Y.; Meng, X.; Zheng, H.; Xu, Y.; Cai, S.; Cai, G.; et al. A study of peritoneal metastatic xenograft model of colorectal cancer in the treatment of hyperthermic intraperitoneal chemotherapy with Raltitrexed. *Biomed Pharmacother* **2017**, *92*, 149-156, doi:10.1016/j.biopha.2017.04.053.
41. Carlier, C.; Laforce, B.; Van Malderen, S.J.M.; Gremonprez, F.; Tucoulou, R.; Villanova, J.; De Wever, O.; Vincze, L.; Vanhaecke, F.; Ceelen, W. Nanoscopic tumor tissue distribution of platinum after intraperitoneal administration in a xenograft model of ovarian cancer. *J Pharm Biomed Anal* **2016**, *131*, 256-262, doi:10.1016/j.jpba.2016.09.004.
42. Lehmann, K.; Rickenbacher, A.; Jang, J.H.; Oberkofler, C.E.; Vonlanthen, R.; von Boehmer, L.; Humar, B.; Graf, R.; Gertsch, P.; Clavien, P.A. New insight into hyperthermic intraperitoneal chemotherapy: induction of oxidative stress dramatically enhanced tumor killing in in vitro and in vivo models. *Ann Surg* **2012**, *256*, 730-737; discussion 737-738, doi:10.1097/SLA.0b013e3182737517.
43. Trepanier, J.S.; Sideris, L.; Lee, L.; Tremblay, J.F.; Drolet, P.; Dube, P. Impact of electrocautery and hyperthermic intraperitoneal chemotherapy on intestinal microvasculature in a murine model. *Int J Hyperthermia* **2016**, *32*, 483-487, doi:10.3109/02656736.2016.1155759.
44. Badrudin, D.; Perrault-Mercier, C.; Bouchard-Fortier, A.; Hubert, J.; Leblond, F.A.; Sideris, L.; Dube, P. Pharmacokinetics and the effect of heat on intraperitoneal pemetrexed using a murine model. *Surg Oncol* **2016**, *25*, 435-440, doi:10.1016/j.suronc.2016.05.014.
45. Badrudin, D.; Sideris, L.; Perrault-Mercier, C.; Hubert, J.; Leblond, F.A.; Dube, P. Comparison of open and closed abdomen techniques for the delivery of intraperitoneal pemetrexed using a murine model. *J Surg Oncol* **2018**, *117*, 1318-1322, doi:10.1002/jso.24960.
46. Piche, N.; Leblond, F.A.; Sideris, L.; Pichette, V.; Drolet, P.; Fortier, L.P.; Mitchell, A.; Dube, P. Rationale for heating oxaliplatin for the intraperitoneal treatment of peritoneal carcinomatosis: a study of the effect of heat on intraperitoneal oxaliplatin using a murine model. *Ann Surg* **2011**, *254*, 138-144, doi:10.1097/SLA.0b013e3182193143.

47. Badrudin, D.; Sideris, L.; Leblond, F.A.; Pichette, V.; Cloutier, A.S.; Drolet, P.; Dube, P. Rationale for the administration of systemic 5-FU in combination with heated intraperitonal oxaliplatin. *Surg Oncol* **2018**, *27*, 275-279, doi:10.1016/j.suronc.2018.05.004.
48. Cianci, S.; Vizzielli, G.; Fagotti, A.; Pacelli, F.; Di Giorgio, A.; Tropea, A.; Biondi, A.; Scambia, G. A novel HIPEC technique using hybrid CO2 recirculation system: intra-abdominal diffusion test in a porcine model. *Updates Surg* **2018**, *70*, 529-533, doi:10.1007/s13304-018-0557-x.
49. Sanchez-Garcia, S.; Padilla-Valverde, D.; Villarejo-Campos, P.; Garcia-Santos, E.P.; Martin-Fernandez, J. Hyperthermic chemotherapy intra-abdominal laparoscopic approach: development of a laparoscopic model using CO2 recirculation system and clinical translation in peritoneal carcinomatosis. *Int J Hyperthermia* **2017**, *33*, 684-689, doi:10.1080/02656736.2017.1302100.
50. Sanchez-Garcia, S.; Padilla-Valverde, D.; Villarejo-Campos, P.; Martin-Fernandez, J.; Garcia-Rojo, M.; Rodriguez-Martinez, M. Experimental development of an intra-abdominal chemohyperthermia model using a closed abdomen technique and a PRS-1.0 Combat CO2 recirculation system. *Surgery* **2014**, *155*, 719-725, doi:10.1016/j.surg.2013.12.005.
51. Padilla-Valverde, D.; Villarejo, P.; Redondo, J.; Oyarzabal, J.; Estella, A.; Palomino, T.; Fernandez, E.; Sanchez, S.; Sanchez, S.; Faba, P.; et al. Laparoscopic cytoreductive surgery and HIPEC is effective regarding peritoneum tissue paclitaxel distribution. *Clin Transl Oncol* **2019**, *21*, 1260-1269, doi:10.1007/s12094-019-02052-8.
52. Tavernier, C.; Passot, G.; Vassal, O.; Allaouchiche, B.; Decullier, E.; Bakrin, N.; Alyami, M.; Davigo, A.; Bonnet, J.M.; Louzier, V.; et al. Pressurized intraperitoneal aerosol chemotherapy (PIPAC) might increase the risk of anastomotic leakage compared to HIPEC: an experimental study. *Surg Endosc* **2020**, *34*, 2939-2946, doi:10.1007/s00464-019-07076-3.
53. Gesson-Paute, A.; Ferron, G.; Thomas, F.; de Lara, E.C.; Chatelut, E.; Querleu, D. Pharmacokinetics of oxaliplatin during open versus laparoscopically assisted heated intraoperative intraperitoneal chemotherapy (HIPEC): an experimental study. *Ann Surg Oncol* **2008**, *15*, 339-344, doi:10.1245/s10434-007-9571-9.
54. Giger-Pabst, U.; Bucur, P.; Roger, S.; Falkenstein, T.A.; Tabchouri, N.; Le Pape, A.; Lerondel, S.; Demtroder, C.; Salame, E.; Ouaissi, M. Comparison of Tissue and Blood Concentrations of Oxaliplatin Administrated by Different Modalities of Intraperitoneal Chemotherapy. *Ann Surg Oncol* **2019**, *26*, 4445-4451, doi:10.1245/s10434-019-07695-z.
55. Ortega-Deballon, P.; Facy, O.; Jambet, S.; Magnin, G.; Cotte, E.; Beltramo, J.L.; Chauffert, B.; Rat, P. Which method to deliver hyperthermic intraperitoneal chemotherapy with oxaliplatin? An experimental comparison of open and closed techniques. *Ann Surg Oncol* **2010**, *17*, 1957-1963, doi:10.1245/s10434-010-0937-z.
56. Ferron, G.; Gesson-Paute, A.; Classe, J.M.; Querleu, D. Feasibility of laparoscopic peritonectomy followed by intra-peritoneal chemohyperthermia: an experimental study. *Gynecol Oncol* **2005**, *99*, 358-361, doi:10.1016/j.ygyno.2005.06.043.
57. Facy, O.; Al Samman, S.; Magnin, G.; Ghiringhelli, F.; Ladoire, S.; Chauffert, B.; Rat, P.; Ortega-Deballon, P. High pressure enhances the effect of hyperthermia in intraperitoneal chemotherapy with oxaliplatin: an experimental study. *Ann Surg* **2012**, *256*, 1084-1088, doi:10.1097/SLA.0b013e3182582b38.
58. Coccolini, F.; Acocella, F.; Morosi, L.; Brizzola, S.; Ghiringhelli, M.; Ceresoli, M.; Davoli, E.; Ansaloni, L.; D'Incalci, M.; Zucchetti, M. High Penetration of Paclitaxel in Abdominal Wall of Rabbits after Hyperthermic Intraperitoneal Administration of Nab-Paclitaxel Compared to Standard Paclitaxel Formulation. *Pharm Res* **2017**, *34*, 1180-1186, doi:10.1007/s11095-017-2132-4.
59. Tang, L.; Mei, L.J.; Yang, X.J.; Huang, C.Q.; Zhou, Y.F.; Yonemura, Y.; Li, Y. Cytoreductive surgery plus hyperthermic intraperitoneal chemotherapy improves survival of gastric cancer with peritoneal carcinomatosis: evidence from an experimental study. *J Transl Med* **2011**, *9*, 53, doi:10.1186/1479-5876-9-53.
60. Tang, L.; Duan, R.; Zhong, Y.J.; Firestone, R.A.; Hong, Y.P.; Li, J.G.; Xin, Y.C.; Wu, H.L.; Li, Y. Synthesis, identification and in vivo studies of tumor-targeting agent peptide doxorubicin (PDOX) to treat peritoneal carcinomatosis of gastric cancer with similar efficacy but reduced toxicity. *Mol Cancer* **2014**, *13*, 44, doi:10.1186/1476-4598-13-44.
